# A Platform for High‐Throughput Assessments of Environmental Multistressors

**DOI:** 10.1002/advs.201700677

**Published:** 2018-01-24

**Authors:** Brian Nguyen, Percival J. Graham, Chelsea M. Rochman, David Sinton

**Affiliations:** ^1^ Department of Mechanical and Industrial Engineering and Institute for Sustainable Energy University of Toronto 5 King's College Road Toronto ON M5S 3G8 Canada; ^2^ Department of Ecology and Evolutionary Biology University of Toronto 25 Wilcocks St Toronto ON M5S 3B2 Canada

**Keywords:** algae, gradient generators, high‐throughput methods, microdevices, multi‐stressors

## Abstract

A platform compatible with microtiter plates to parallelize environmental treatments to test the complex impacts of multiple stressors, including parameters relevant to climate change and point source pollutants is developed. This platform leverages (1) the high rate of purely diffusive gas transport in aerogels to produce well‐defined centimeter‐scale gas concentration gradients, (2) spatial light control, and (3) established automated liquid handling. The parallel gaseous, aqueous, and light control provided by the platform is compatible with multiparameter experiments across the life sciences. The platform is applied to measure biological effects in over 700 treatments in a five‐parameter full factorial study with the microalgae *Chlamydomonas reinhardtii*. Further, the CO_2_ response of multicellular organisms, *Lemna gibba* and *Artemia salina* under surfactant and nanomaterial stress are tested with the platform.

## 1 Introduction

Globally, communities are coping with warming in combination with increased CO_2_ levels, occurring concurrently with local environmental stressors from synthetic chemicals.^1^, ^2^, ^3^, ^4^ The challenge of predicting biological responses stems from needing to measure impacts from multiple variables at once, including changes in aqueous nutrients and toxins, gasses, light, and temperature.^5^, ^6^, ^7^, ^8^, ^9^ Although multiple stressors are the norm, most studies focus on effects from a single stressor.^10^ Microcosms and mesocosms allow both a high degree of control over parameters, and parallelization.^11^, ^12^ Unfortunately, with current microcosm‐based techniques, full factorial experiments involving multiple variables (e.g., temperature, light, gas, chemical, and a nanomaterial pollutants) are challenging and generally limits replication, making it difficult to truly measure an effect—once the number of experimental treatments reaches the 100's—typical of multiparameter studies.^5^, ^7^

While local stressors (e.g., point source pollution, resource extraction) vary geographically, stress associated with elevated CO_2_ is global. Because elevated atmospheric CO_2_ is ubiquitous and a particularly important environmental driver,^5^, ^13^ understanding biotic responses to the continually increasing CO_2_ levels, in combination with other drivers,^5^, ^6^, ^14^ is key to understanding impacts of global change. In addition, it helps more realistically predict future atmospheric CO_2_ concentrations—as affected by primary productivity feedback.^15^, ^16^ At the organism level, responses to elevated CO_2_ concentrations are multifaceted, especially when combined with other environmental parameters.^5^, ^16^ At the community level, this complexity is compounded by trophic interactions.^17^ Unfortunately, examining more than a few CO_2_ concentrations in experiments is not feasible with conventional techniques, since traditional gas handling requires costly and elaborate equipment even for a few distinct concentration levels.^7^

In addition to CO_2_, nitrogen, phosphate, temperature, and light are key environmental parameters.^5^ Moreover, an experimenter may want to measure impacts from climate change in concert with other anthropogenic stressors, such as a chemicals or nanomaterials. A full factorial sweep of 32 levels of CO_2_, representing future climate scenarios with CO_2_ typical supersaturation in lakes^18^, ^19^ and soils,^20^ in concert with two levels of each of the other aforementioned parameters with three replicates would require over 1500 experimental units. Although models can expand predictive power from smaller data sets, they may overlook interactive effects and thus provide limited mechanistic insight.^5^, ^7^ In the context of the complex environmental questions, all approaches benefit from the ability to aquire larger experimental data sets.

Lab‐on‐a‐chip microdevices^21^, ^22^, ^23^, ^24^, ^25^, ^26^, ^27^ have shown promise in overcoming the throughput barriers owing to their exceptional liquid handling capabilities.^23^, ^28^ While microdevices provide a route to meeting the high‐throughput requirements, current microdevices lack the capability, versatility, usability, and scalability required to address the throughput challenge presented by multistressor global change.^29^, ^30^ Diffusion based microfluidic gradient generators can provide a range of concentrations by leveraging the ability of small molecules to diffuse between a source and a sink through a bulk material, typically polydimethylsiloxane (PDMS)^31^ or hydrogel.^32^ However, current gradient generators are limited in size due to the relatively small rate of diffusion in PDMS and hydrogels. As a result, these platforms can accommodate only a few (<10) distinct chemical environments in very small culture volumes.^32^, ^33^, ^34^, ^35^, ^36^ Thus, in their current state, existing platforms are incapable of producing large‐scale gas gradients required to produce sufficient distinct levels needed to resolve the impacts of CO_2_ concentration on cells (much less multicellular biota) and allow simultaneous control of multiple variables.^32^ Moreover, microdevices currently face practical barriers to widespread adoption. Specifically, scaling microfabrication remains a challenge, a limitation compounded by the single‐use nature of microdevices and the need for specialized equipment to operate.^30^

Here, we developed a technique that is high‐throughput and uses microcosms to measure impacts to individuals and biological communities from CO_2_ in combination with multiple stressors (e.g., temperature, light, nutrient availability, chemical pollutants, particulate pollutants). We leverage the uniquely high rate of diffusion‐dominated mass transport in aerogels to create a gradient generator to sweep multiple CO_2_ concentrations. We combine our aerogel‐based gradient generator with established automated liquid handling and light control with liquid crystal display (LCD) projection to produce a platform that enables large factorial experiment designs involving microorganisms, as well as small multicellular biota. We apply this approach to multiparameter experiments with three kinds of model organisms. With the model microorganism *Chlamydomonas reinhardtii*, we demonstrate that our platform can obtain a full‐factorial dataset consisting of five environmental parameters, 768 treatments, entailing ≈3000 experimental units. We also demonstrate compatibility with multicellular organisms, *Lemna gibba* and *Artemia salina*.

## 2 Results and Discussion

### 2.1 Aerogel Gradient Generator Performance

**Figure 1:**
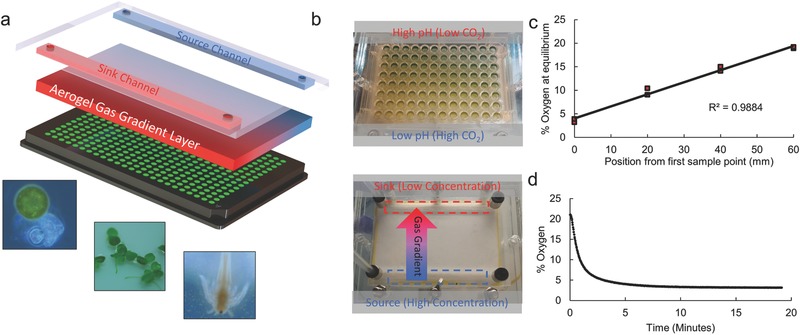
a) Expanded schematic of the aerogel‐based gas gradient generating method with insets showing model organisms compatible with the setup, from left to right: microalgae, macrophytes, and crustaceans. b) pH‐indicator visualization of the CO_2_ gradient in a clear 96‐well plate, at increasing concentrations of CO_2_ the bromothymol blue‐based pH indicator solution turns from blue to yellow due to increasing CO_2_‐induced acidity. c) Measurement of oxygen concentration at equilibrium at points along an oxygen gradient, markers represent measurements on two separate devices of identical design. The linearity of the gradient and the temporal diffusion profile confirm that the mass transport of gas in the aerogel can be accurately described solely using Fick's laws. d) Measured concentration of a sample point over time after applying gas concentrations showing ≈15 min to reach equilibrium, consistent with a diffusion coefficient of ≈2 × 10^−2^ cm^2^ s^−1^ or one‐tenth of that of open air.

**Figure** **1** shows our aerogel‐based gas concentration gradient generator. The source–sink configuration (Figure 1a) of our device is designed to create linear gas concentration gradients across a well plate‐sized area (≈7 cm by 10 cm), between the source and sink channels. Aerogels are open‐cell, nanoporous foams, with extremely high porosity. The open‐cell structure and high porosity of aerogels enables the rate of diffusion in aerogels to be about one‐tenth of that in open air— ≈1000 times higher than the rate of diffusion in PDMS or water.^37^ Furthermore, the nanoporosity of aerogels allows mass transport to be consistently dominated by diffusion with negligible advective transport, even in the gas phase allowing predictable equilibrium concentration profiles (details in the Supporting Information).^38^

To confirm the linearity and equilibrium time of a gas concentration gradient (Figure 1c), four sampling points were measured across a generated oxygen gradient (Figure S1, Supporting Information). The response time of our gradient generator is approximately three orders of magnitude faster (Figure 1d) than existing microfluidic gradient generators^38^ as a direct consequence of the high diffusivity in aerogels. This rapid response time enables the creation of centimeter‐scale concentration gradients on convenient experimental setup timescales (≈15 min). In contrast, such a gradient would take ≈7 d with hydrogel or PDMS‐based gradient generators. The ability to generate CO_2_ gradients over well plates was confirmed using a bromothymol blue‐based pH indicator solution in 96‐well plates (Figure 1b). The gradient can be applied across any well plate, with different organisms and liquid‐phase compositions (Figure S2, Supporting Information).

The high rate of diffusion has the added benefit of being robust against perturbation by cell culture. In contrast to slower diffusion‐based gradient generators, our numerical simulations show that the gradient in the aerogel device is not significantly perturbed (<2.5%) even if a 1536‐well plate was filled with dense cultures all drawing CO_2_ at maximal rates (Figure S3 and Table S1, Supporting Information). In contrast, a modest culture would perturb a traditional diffusion‐based gradient generator, even if the set‐up time could be tolerated (Figure S3 and Table S1, Supporting Information). Further, as the aerogel gradient generator works solely by gas‐phase diffusion, we expect it to be compatible with the full range of biologically relevant gas streams without modification allowing application to other life science or chemical research where gas concentrations are relevant including: small animal and cellular chemotaxis^29^, ^39^ and stem cell culture conditions.^23^, ^40^ The gradient generator can be used for an indefinite number of experimental runs and only the well plate needs to be replaced. Additionally, our approach is widely compatible with established liquid handling techniques.

### 2.2 Full Factorial Experiment with Microalgae

**Figure 2:**
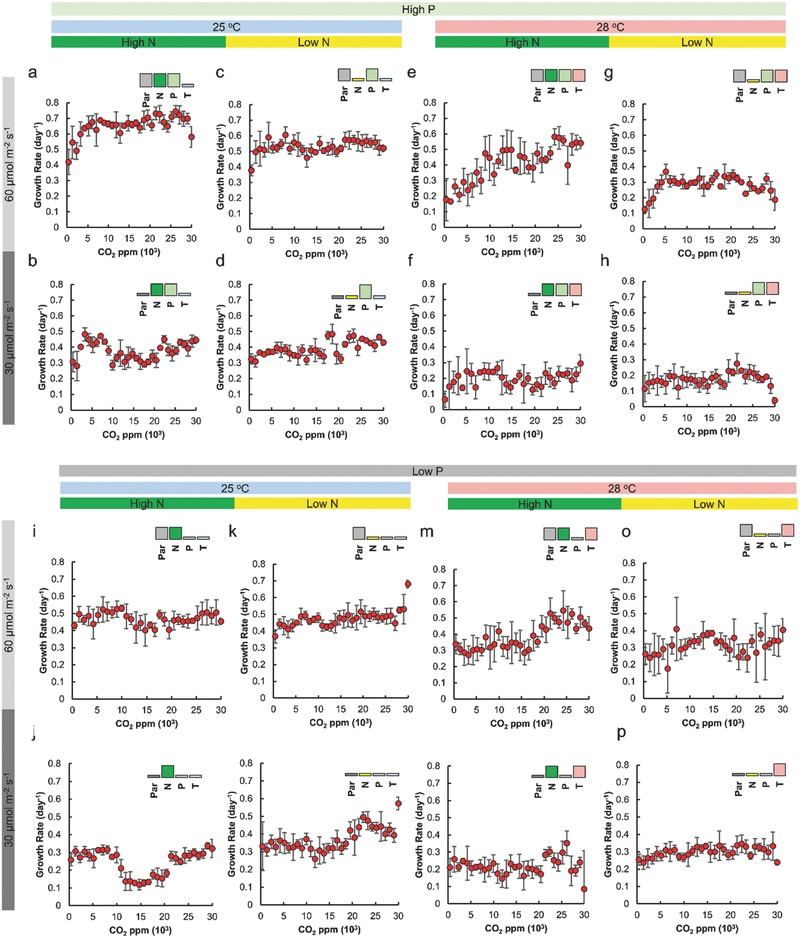
CO_2_ response curves for *Chlamydomonas reinhardtii* showing the combined effect of CO_2_ (0–30 000 ppm), temperature (25 and 28 °C), irradiance (30 µmol m^−2^ s^−1^ and 60 µmol m^−2^ s^−1^), nitrogen (9.4 and 0.94 × 10^−3^ m NH_4_Cl starting concentrations) and phosphorus (13.5 × 10^−3^ and 2.7 × 10^−3^ m total P starting concentrations). Growth rates were calculated for 4 d of growth. In all cases *n* = 4 for each condition with the exception of a, b, c, e, p where *n* = 3 to accommodate “blank” wells. Error bars represent standard deviations in all cases.

The data from the *C. reinhardtii* experiment represents 768 unique treatments with four replicates each except for the treatments with standard media and high light to accommodate a “blank” column on the well plates, for a total of ≈3000 experimental units (**Figure** **2**; Figure S5, Supporting Information). Under control conditions (25 °C, nutrient replete, light replete), the influence from CO_2_ on the growth of *C. reinhardtii* follows Monod kinetics, saturating at ≈5000 ppm CO_2_ (Figure 2a). This response is consistent with the literature.^5^, ^41^, ^42^, ^43^

Fitting a linear model^44^ reveals significant interactions between the parameters we tested. Light intensity and CO_2_ show strong main effects (**Table** **1**). This can be explained by the key role of light as the energy supply in photosynthesis and CO_2_ as the principle substrate, allowing it to compensate for lower concentrations of other resources by facilitating active transport.^16^ However, all parameters are significantly involved in the growth response via interactions with each other.

**Table 1:** Linear model summary, coefficients are linear regression coefficients for a model where *C* is in percent CO_2_, *P* is phosphorus (mM), *N* is nitrogen in (mM), *L* is light in µmol m^−2^ s^−1^, and *T* is warming above 25 °C. Example calculation for 30 000 ppm CO_2,_ 13.5 (mM) *P*, 9.4 (mM) *N*, 60 µmol m^−2^ s^−1^ and 0 °C warming

Term	Coefficient	Contribution at example value	Std Error	*t* Ratio	*p*‐value
*T*	−5.096E‐02	0	1.104E‐03	−4.617E+01	<0.001
*P*	6.004E‐03	8.106E‐02	3.125E‐04	1.921E+01	<0.001
*N*	2.696E‐03	2.534E‐02	3.913E‐04	6.890E+00	<0.001
*L*	5.492E‐03	3.295E‐01	1.102E‐04	4.983E+01	<0.001
*C*	3.140E‐06	9.421E‐02	1.904E‐07	1.649E+01	<0.001
(*T*‐1.49843)*(*P*‐5.35657)*(*L*‐44.0765)*(*C*‐15218.1)	3.426E‐09	−9.839E‐03	1.580E‐09	2.168E+00	0.030248
(*T*‐1.49843)*(*P*‐5.35657)*(*C*‐15218.1)	5.645E‐08	−1.018E‐02	2.355E‐08	2.397E+00	0.016605
(*T*‐1.49843)*(*P*‐5.35657)	−2.409E‐03	2.940E‐02	2.061E‐04	−1.169E+01	<0.001
(*T*‐1.49843)*(*N*‐5.08025)*(*P*‐5.35657)*(*L*‐44.0765)*(*C*‐15218.1)	−8.567E‐10	1.063E‐02	3.773E‐10	−2.271E+00	0.023234
(*T*‐1.49843)*(*N*‐5.08025)*(*P*‐5.35657)*(*L*‐44.0765)	−2.076E‐05	1.742E‐02	3.222E‐06	−6.443E+00	<0.001
(*T*‐1.49843)*(*N*‐5.08025)*(*P*‐5.35657)	−4.128E‐04	2.176E‐02	4.802E‐05	−8.596E+00	<0.001
(*T*‐1.49843)*(*N*‐5.08025)*(*L*‐44.0765)	5.834E‐05	−6.013E‐03	1.737E‐05	3.359E+00	<0.001
(*T*‐1.49843)*(*N*‐5.08025)*(*C*‐15218.1)	1.130E‐07	−1.081E‐02	2.980E‐08	3.792E+00	<0.001
(*T*‐1.49843)*(*N*‐5.08025)	3.932E‐03	−2.545E‐02	2.597E‐04	1.514E+01	<0.001
(*T*‐1.49843)*(*L*‐44.0765)*(*C*‐15218.1)	6.098E‐08	−2.151E‐02	8.433E‐09	7.231E+00	<0.001
(*T*‐1.49843)*(*L*‐44.0765)	−4.712E‐04	1.124E‐02	7.331E‐05	−6.428E+00	<0.001
(*P*‐5.35657)*(*L*‐44.0765)*(*C*‐15218.1)	6.994E‐09	1.341E‐02	2.366E‐09	2.957E+00	0.003136
(*P*‐5.35657)*(*C*‐15218.1)	9.337E‐08	1.124E‐02	3.593E‐08	2.599E+00	0.0094
(*N*‐5.08025)*(*P*‐5.35657)*(*L*‐44.0765)*(*C*‐15218.1)	1.899E‐09	1.573E‐02	5.731E‐10	3.314E+00	<0.001
(*N*‐5.08025)*(*P*‐5.35657)*(*L*‐44.0765)	3.486E‐05	1.953E‐02	4.857E‐06	7.177E+00	<0.001
(*N*‐5.08025)*(*P*‐5.35657)	2.763E‐04	9.719E‐03	7.268E‐05	3.802E+00	0.000147
(*N*‐5.08025)*(*L*‐44.0765)*(*C*‐15218.1)	1.573E‐08	1.600E‐02	3.017E‐09	5.215E+00	<0.001
(*N*‐5.08025)*(*L*‐44.0765)	3.205E‐04	2.204E‐02	2.611E‐05	1.227E+01	<0.001
(*L*‐44.0765)*(*C*‐15218.1)	4.203E‐08	9.892E‐03	1.262E‐08	3.329E+00	<0.001
Intercept	7.160E‐02	7.160E‐02	3.002E‐02	4.483E+01	<0.001
Growth Rate Estimate		7.260E‐01			

Since rising temperatures occur concurrently with rising CO_2_ levels, the combined effect of rising CO_2_ and temperature is particularly important. Temperature influences how primary producers respond to rising O_2_ by decreasing gas solubility, increasing metabolism, increasing photorespiration,^45^ and modulating inorganic carbon uptake mechanisms.^46^ The combination of these mechanisms make the CO_2_ response difficult to predict.^5^, ^15^, ^45^, ^46^, ^47^ Interestingly, all significant interactions that involve CO_2_ and temperature have positive coefficients in the linear model which indicates that the negative effect of increased warming is decreased as CO_2_ concentrations increase. This could point to higher CO_2_ levels offsetting increased photorespiration^45^ and the lower solubility of CO_2_ at higher temperatures (1.45 g L^−1^ at 25 °C vs 1.33 g L^−1^ at 28 °C). This effect suggests that CO_2_ supersaturation in lakes^18^, ^19^ and soils^20^ may help algae cope with elevated temperatures.

### 2.3 Our Platform is Compatible with Multicellular Plants

We tested compatibility of our platform with multicellular organisms starting with the model plant, *L. gibba*. We demonstrated the compatibility of our platform by tracking the effect of CO_2_ on growth with and without pollutants (linear alkylbenzene sulfonate (LAS) or titania nanoparticles). However, we should note that the relatively small wells of our device limit the size of plants that can be used due to the confinement responses that are possible. While several model plants such as *Lemna* 48 and *Arabpidopsis* 21 are known to be compatible with low‐volume culture, an experimenter should verify our platform's suitability for experiments with other plants.

**Figure 3:**
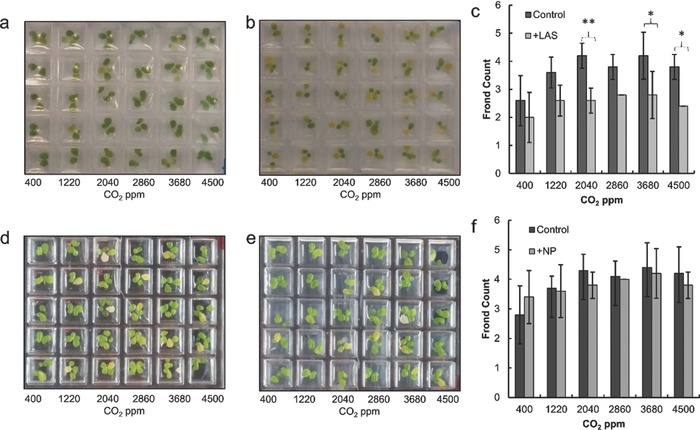
Images of *Lemna gibba* cultured for 7 d a) without and b) with the surfactant LAS, a common pollutant. c) Mean frond counts of *Lemna gibba* after 7 d with and without LAS (*n* = 5 for treatments with LAS, error bars represent standard deviations) (Images of *Lemna gibba* cultured for 7 d d) without and e) with titania nanoparticles. f) Mean frond counts of *Lemna gibba* after 7 d with and without titania nanoparticles (*n* = 5 for treatments with nanoparticles, error bars represent standard deviations). represents *p* < 0.05, ** represents *p* < 0.01 and with a two‐factor ANOVA with Tukey's HSD.

We observed that the growth response of *L. gibba* to CO_2_ concentrations follows Monod kinetics, with saturation occurring between 1200 and 2000 ppm in standard media, in agreement with the literature.^49^ However, pollutants can alter this CO_2_ response. LAS inhibits growth under all tested CO_2_ conditions and results in shrunken and discolored fronds— indicating poor health (**Figure** **3**b). Moreover, under LAS stress, CO_2_ stimulation of growth is effectively suppressed (Figure 3c). At above 2000 ppm CO_2_, we observed significantly fewer fronds compared to the treatment without LAS. We postulate that the increased LAS inhibition at high CO_2_ levels is due to reduced chlorophyll concentration,^50^ apparent from the discolored fronds. With lower chlorophyll levels, light harvesting is less efficient causing growth to become more light limited than carbon limited. In short, the LAS stress may weaken the negative climate feedback effect of increasing primary production by inhibiting light harvesting. In contrast to LAS, significant inhibition by titania nanoparticles (used as a pigment for industrial and consumer applications) was not observed at any individual CO_2_ level, consistent with existing literature.^51^ Moreover, titania nanoparticles did not cause any obvious discoloration or deformation of the fronds. Low levels of titania nanoparticles are considered relatively harmless and can even have stimulatory effects on plant growth by enhancing chloroplast activity under current atmospheric CO_2_ conditions.^51^

### 2.4 Our Platform is Compatible with Small Animals

**Figure 4:**
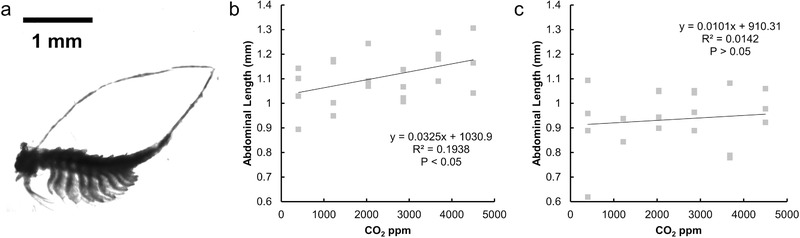
a) Representative image of an *Artemia salina* sub‐adult grown from a nauplius. b) Growth response to CO_2_ of *Artemia salina* cultured with live *Duniealla salina* in brine without titania nanoparticles. Black circles represent mean abdominal lengths; grey squares represent individual abdominal lengths; error bars represent standard deviations. c) Growth response to CO_2_ of *Artemia salina* cultured with live *Duniealla salina* cultured in brine with titania nanoparticles.

Elevated CO_2_ levels are expected to have a detrimental effect on brine shrimp nauplii growth due to acidification.^52^ *Duniealla salina* is part of the typical diet of *A. salina* and therefore growth stimulation by elevated CO_2_ in *D. salina* could have a positive effect on the growth of *A. salina* nauplii despite acidification.^53^ Using our platform, we studied these direct and indirect effects for six different CO_2_ concentrations (**Figure** **4**). Our results show that a moderate positive effect (*P* = 0.04 calculated from Pearson's correlation coefficient) of elevated CO_2_ is present at the early stages of development where growth (measured by abdominal length) increased likely due to greater food availability (Figure 4b). When grown without *A. salina, D. salina* shows increased growth with increasing CO_2_ (Figure S13, Supporting Information). However, this effect loses significance when titania nanoparticles (Figure 4c), which impede the growth of microalgae,^54^ are introduced.

## 3 Conclusion

We developed a simple, but powerful platform, leveraging rapid and controlled gas diffusion through aerogels in combination with spatial light control, for parallel screening of biological responses to multiple experimental parameters in microcosms. The ability to rapidly investigate the effect of multiple parameters is a step toward overcoming the throughput barrier to performing detailed full factorial studies that capture the complexity of multiple environmental stressors.

Our platform enables the observation of phenomena that have implications for the role of primary productivity in the context of rising CO_2_ levels with unprecedented throughput. Our method allows for investigation of a wide range of CO_2_ conditions in combination with other environmental parameters and is compatible with both microorganisms and small multicellular organisms. We expect that our approach will be applicable in furthering our understanding of the ecological impacts of climate change together with other environmental stressors.

## 4 Experimental Section

*Aerogel‐Based Gradient Generator Construction*: Hydrophobic aerogel monoliths (Aerogel technologies P‐AT.A.X103, medium density, large panels) were purchased from Aerogel Technologies Inc. These aerogels had > 80% porosity^55^ and pores averaging ≈50 nm. The ideal aerogel for this application would have an interconnected, low tortuosity pore structure with maximum void space to maximize diffusion and small pore sizes to minimize advective mass transport. A high tortuosity porous structure was not desirable since it creates a longer effective diffusion path. The aerogel monoliths were cut to size with a fine‐toothed saw, sanded flat with 320 grit sandpaper, and thinned to a thickness of ≈6 mm. Care was taken not to contact the aerogel with solvents that would collapse the pore structure and hinder gas diffusion. The experimental apparatus consisted of a standard well plate and aerogel sandwiched between an acrylic back plate and an acrylic layer with source and sink channels 6 mm tall and 6 mm wide spaced 6.8 cm apart, center to center (6.2 cm edge to edge) (Figure S6b, Supporting Information). All acrylic components were fabricated using a CO_2_ laser cutter (Universal Laser Systems M‐360). Acrylic parts were bonded in a Carver hot press. The open areas of the aerogel were sealed with polyethylene terephthalate (PET) tape to ensure no‐flux conditions where desired (i.e., the source edge and the edges orthogonal to the channels). A silicon gasket was used to ensure a seal between the aerogel and the layer with the source and sink channels. While silicon is gas permeable, it did not permit advective mass transport. Clamping force was applied using ¼‐20 machine screws and wing nuts. The highest and lowest concentrations in the CO_2_ gradient were set by adjusting the concentration of CO_2_ in the gas flowing in the source and sink channel respectively. All gas inlets were maintained at constant pressure using pressure regulators.

For the oxygen measurements, a custom acrylic manifold (Figure S1, Supporting Information) was used to position a needle‐type optode (Presens Precision Sensing GmbH) at four sample points spaced at a pitch of 20 mm. The optode measurements were conducted on two devices using ≈12 mm thick aerogels (Aerogel technologies P‐AT.A.X103 medium density, tiles). The optodes were punctured through a custom silicon sheet that acted as a septum. For the pH indicator tests, a saturated bromothymol blue solution was adjusted to show blue at room CO_2._ A 96‐well clear plate was filled with 200 µL per well of the bromothymol blue solution and interfaced with the gradient generator. The source channel in the device used for experiments was provided with CO_2_‐enriched air and the sink channel with plain air. This test was also conducted with a methyl red indicator solution (Figure S11, Supporting Information). It was ensured that CO_2_ consumption did not affect the gradient by conservatively modeling the gradient with CO_2_ consumption in COMSOL Multiphysics (Figure S3, Supporting Information).

*Projector Setup*: The projector was mounted at a tilt ≈15° from the vertical and manually aligned with the well plate to ensure that there was no spatial bias in light intensity. The absence of spatial bias was confirmed by measuring light intensities on both sides of a well plate. The light output from the projector was periodically monitored using a quantum meter to ensure that the light intensity did not decrease due to a worn lamp. If the light intensity was found to be low, the lamp was changed. Cultures were illuminated from the bottom of the well plate since the top of the well plate was occupied by the opaque gas gradient generator. However, the experiments were run with the well plate positioned upside down to allow illumination from above. Black‐walled well plates were chosen to eliminate light cross‐talk between wells (Figure S12, Supporting Information).

*Spatial Light Control*: An Epson EX3220 LCD projector was used as a light source (Figure S4, Supporting Information) which allowed high‐resolution spatial control of light intensity by adjusting pixel grey values. Light intensities were measured using a quantum meter (Li‐COR), incident to the acrylic backplate.

*C. reinhardtii Growth Experiments: C. reinhardtii* wild‐type strain CC‐124 was obtained from the Chlamydomonas Resource Centre (University of Minnesota, St. Paul, MN) and maintained in Sueoka's high salt medium^56^ (HSM) at 25 °C and ≈30 µmol m^−2^ s^−1^ in several 250 mL culture flasks. KCl was used to ensure all experimental media had the same salinity.^5^, ^57^ *C. reinhardtii* was particularly suited to this demonstration because it is a widely studied model alga and it is known to be amenable to small scale culture.^5^, ^32^ Variations of HSM with either 0.675, 2.7, or 13.5 × 10^−3^ m phosphorus and 0.94 or 9.4 × 10^−3^ m nitrogen were used as experimental media. Prior to experiments, cultures were acclimated for at least 7 d in the same media composition for the experimental runs in 250 mL culture flasks.^5^ All media was sterile filtered with 0.22 µm vacuum filtration units prior to use.

Experiments were conducted on black‐walled 1536‐well plates for five‐parameter experiments sealed with a breathable membrane^58^, ^59^, ^60^ to reduce evaporation and prevent cross‐contamination (Diversified Biotech Breathe‐easy). The membrane also acted as a gasket to interface the well plate with the aerogel. Microalgal cultures were loaded into the well plates using a Perkin Elmer Flexdrop IV reagent dispenser set to 9 µL per well for 1536‐well plates after resuspension in fresh experimental media. The volumes were chosen based on previously reported experience with *C. reinhardtii* experiments conducted in the same well plate formats.^59^

The well plates were interfaced with the aerogel gradient generator by sandwiching the well plate between a transparent poly(methyl methacrylate) (PMMA) backplate and the aerogel. The source channel was provided with a continuous gas flow of 30 000 ppm CO_2_ and the sink channel was provided a continuous gas flow of 400 ppm CO_2_ to provide a CO_2_ concentration gradient from 400 to 30 000 ppm. The CO_2_ concentrations were chosen to represent the range of concentrations in lakes and soils—which were typically supersaturated with CO_2_.^18^, ^19^ The well plate was aligned with the first row of wells ≈2 mm outside of the sink channel. The experiments were conducted over 4 d in batch. Each treatment was replicated four times each except for the treatments with standard media and high light which was replicated three times to accommodate a “blank” column on the well plates. One 1536 well‐plate was used for each temperature level. Due to the relatively small liquid volumes and high surface area to volume ratios in 1536‐well plates the gas inlets were hydrated by bubbling through deionized water upstream of the gradient generator to further prevent evaporation. A resistive heater coupled to a temperature controller was used to set experimental temperatures. Fans were used to ensure an even temperature distribution throughout the experimental apparatus.

Optical density at 750 nm was measured using a BMG Pherastar FS plate reader. Prior to optical density measurements the well plates were vortex mixed and centrifuged to suspend the cells and eliminate bubbles. Wells containing bubbles after centrifugation were excluded from the analysis, these wells were relatively rare—consisting of fewer than 1% of the total wells used. For optical density measurement, the breathable sealing film was replaced with an optical‐grade sealing film (Greiner VIEWseal). Growth rate was calculated using the following formula^5^

(1) $$\mathrm{Growth}\;\mathrm{Rate}=\frac{\ln({\mathrm{OD}}_{2})-\ln({\mathrm{OD}}_{1})}{t_{2}-t_{1}}$$

*L. gibba Growth Experiments: L. gibba* was obtained from the Canadian Phycological Culture Collection (University of Waterloo) and maintained on Hoagland's media at 25 °C and ≈30 µmol m^−2^ s^−1^. *L. gibba* experiments were conducted in custom bottomless 60‐well plates fabricated from PMMA sealed with a breathable membrane on both sides (Diversified Biotech Breathe‐easy). A custom well plate was used to obtain a suitable well size for culturing *L. gibba*. Each well of the custom well plate was initially loaded with a single plant with a pair of fronds. An initially 2 fronds with 500 µL of experimental medium were loaded in each well. The well plates were interfaced with the aerogel gradient generator by sandwiching the well plate between a PMMA backplate and the aerogel, as with the microalgae experiments.

The aerogel gradient generator was used to apply a CO_2_ concentration gradient of six levels from 400 to 4500 ppm by providing gas streams of 400 ppm and 4500 ppm in the sink and source channel respectively. Continuous light at 60 µmol m^−2^ s^−1^ was provided over the course of the experiment. For cases with LAS stress 25 mg L^−1^ sodium dodecylbenzenesulfonate was added at the beginning of the experiment. For cases with titania nanoparticles 100 mg L^−1^ of 25 nm titania particles (Evonik Aeroxide) were added to the growth media. Each treatment was replicated five times. No media changes were conducted during the experiment. An endpoint frond count was taken after 7 d. Heavily discolored fronds were excluded from the count.^61^

*A. salina Growth Experiments: A. salina* cysts were obtained from Brine Shrimp Direct. *A. salina* cysts were incubated in f/2 nutrient enriched brine (a 6% sea salts solution)^62^ for 24 h to hatch. *Duniellia salina* (UTEX 1644) was obtained from the UTEX Culture Collection of Algae and maintained in f/2 nutrient enriched brine at 25 °C in ambient CO_2_, and ≈30 µmol m^−2^ s^−1^ in several 250 mL culture flasks. Freshly hatched nauplii were individually loaded into 48‐well plates using a micropipette with wide‐orifice tips along with a 1 mL suspension of live *D. salina* (OD_750_ 0.03 measured in a 1 cm cuvette) in f/2 nutrient enriched brine as a food source. A temperature of 25 °C was maintained and continuous light with an intensity of 80 µmol m^−2^ s^−1^ was provided with an light‐emitting diode (LED) lighting panel (spectrum in the Supporting Information). The projector setup was not used for the *Artemia* experiment since it only involved a single light condition. After 7 d, the animals were euthanized by heating to 40 °C, allowed to settle on the bottom of the well plate, and imaged on a microscope. Abdominal length measurements were made manually in ImageJ from the microscope images (Figure S10, Supporting Information). The occasional (less than 10% of the animals) animal that floated to the surface, hampering imaging, upon death was excluded from the analysis. For the experiments with titania nanoparticles, 100 mg L^−1^ 25 nm titania nanoparticles were added to the brine. No media changes were conducted during the experiment.

*Statistics*: All statistical modeling and testing were done in RStudio using R version 3.4.1 or JMP. For the *C. reinhardtii* experiment, a linear model^44^ was fit to the data in JMP. For the *L. gibba* experiments two‐factor ANOVAs with Tukey's HSD post‐hoc testing was conducted. For the *A. salina* experiment, linear regressions were performed. Data were considered statistically significant at *p* < 0.05.

## Conflict of Interest

The authors declare no conflict of interest.

## Supporting information

Supplementary
