# Supplementary material for: A Platform for High‐Throughput Assessments of Environmental Multistressors
Source: Adv Sci (Weinh). 2018 Jan 24;5(4):1700677. doi: 10.1002/advs.201700677 (PMC5908365; doi:10.1002/advs.201700677)
Supplement: Supplementary file 1 — Supplementary [file ADVS-5-1700677-s001.pdf]

Copyright WILEY-VCH Verlag GmbH & Co. KGaA, 69469 Weinheim, Germany, 2018.

Supporting Information

for *Adv. Sci.,* DOI: 10.1002/advs.201700677

A Platform for High-Throughput Assessments of Environmental Multistressors

*Brian Nguyen, Percival J. Graham, Chelsea M. Rochman, and David Sinton**

A Platform for High-Throughput Assessments of Environmental Multi-stressors

Brian Nguyen[a], Percival J Graham[a], Chelsea M Rochman[b] and David Sinton[a]

[a]*Department of Mechanical and Industrial Engineering and Institute for Sustainable Energy, University of Toronto, 5 King's College Road, Toronto, ON, Canada, M5S 3G8.*

[b]*Department of Ecology and Evolutionary Biology, University of Toronto, 25 Wilcocks St, Toronto, ON, Canada, M5S 3B2*

Supporting Information

Supplementary Equation 1:

$$(1)\ \frac{dC(x,t)}{dt} = D\,\frac{d^2C(x,t)}{dx^2}$$

At equilibrium:

$$(2)\ \frac{dC(x)}{dt} = 0$$

With

$$(3)\ C(0) = C_1 \ \&\ C(L) = C_2$$

$$(4)\ C(x) = Kx + C_1$$

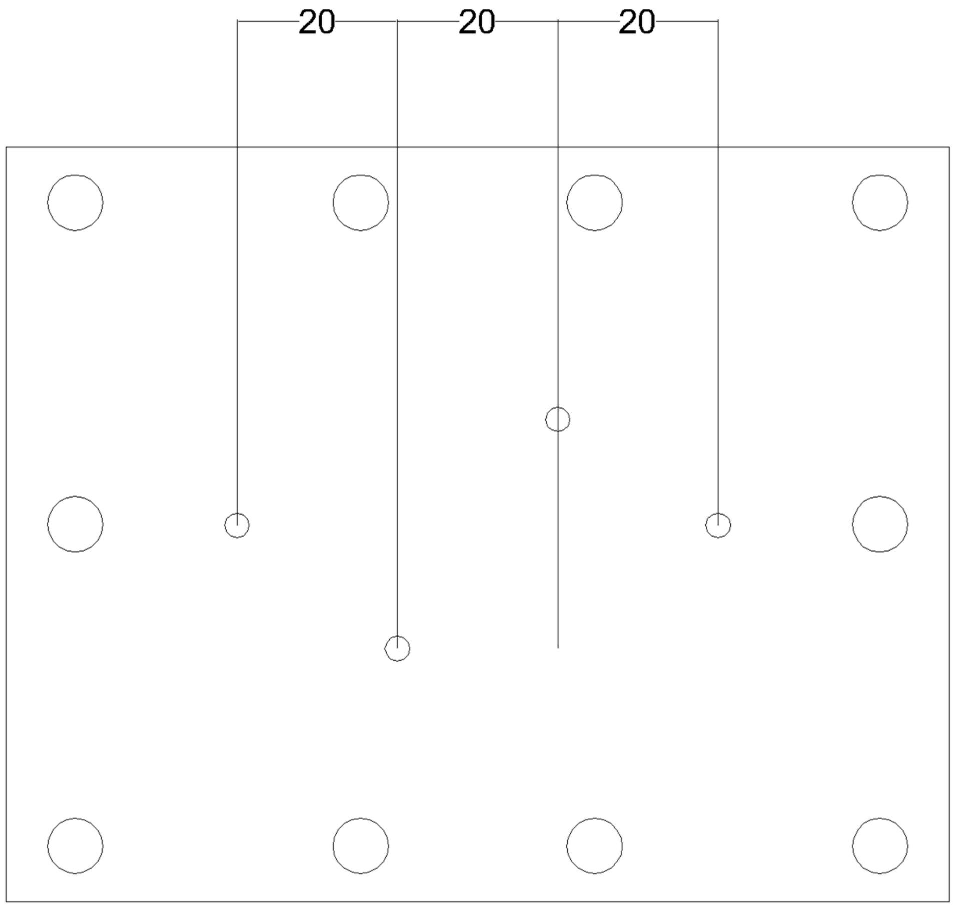

Figure S1: (a) Layout of oxygen sensing points. Oxygen measurements were taken from the four staggered points. The points were equally spaced in the horizontal direction. Distance was measured from the first point on the left (the low oxygen side). The total distance between the all points was 6 cm (b) The points were situated between two parallel channels on the opposite side of a 1.5 cm thick aerogel. Units are in mm.

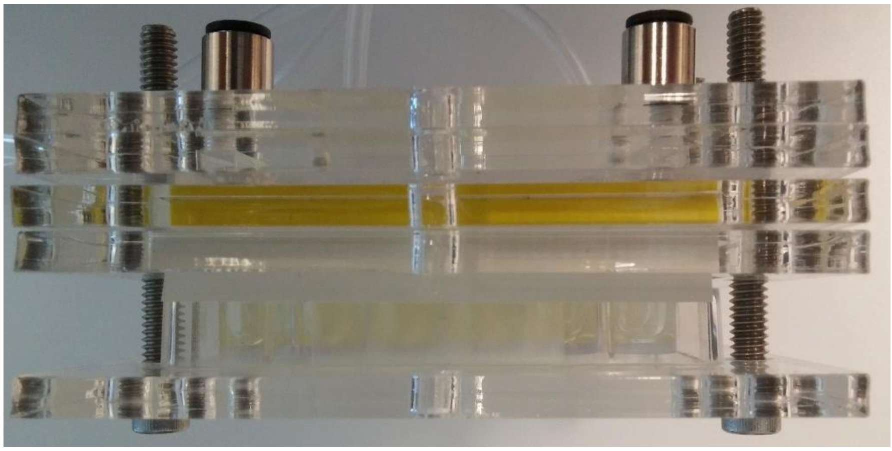

Figure S2: Assembled gas gradient generator with a 48-well plate. The edges of the aerogel are sealed with the yellow Polyethylene terephthalate tape. The top plate, containing the channels, was fabricated out of 2 layers of 6 mm thick acrylic. Acrylic ring spacers were used to align the well plate and the aerogel. The bottom plate was fabricated from 1 layer of 6 mm PMMA.

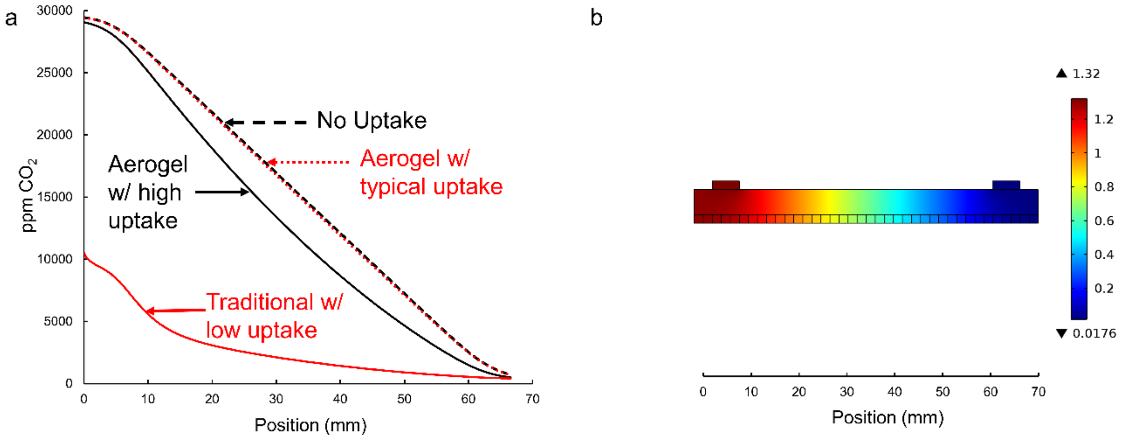

Figure S3: COMSOL Multiphysics® simulation of the effects of CO_2 uptake by cultures (a) The effect of CO_2 uptake on the gradient profile (b) Visual representation of the gradient under typical CO_2 uptake with the aerogel gradient generator according to the simulation, units on the color scale are in mol/m^3.

Supporting Table 1: Parameters used for the COMSOL simulations. We assume that Chlorophyll is 5% of the dry weight[1].

	Cell density (mg dry weight/mL)	*Photosynthetic rate (μmol mg Chl^{-1} hr^{-1})*
Aerogel (no consumption)	0	0
Aerogel (typical consumption)	0.08	100[2]
Aerogel (high consumption)	0.4	400[2,3]
Traditional (low consumption)	0.02	50[2]

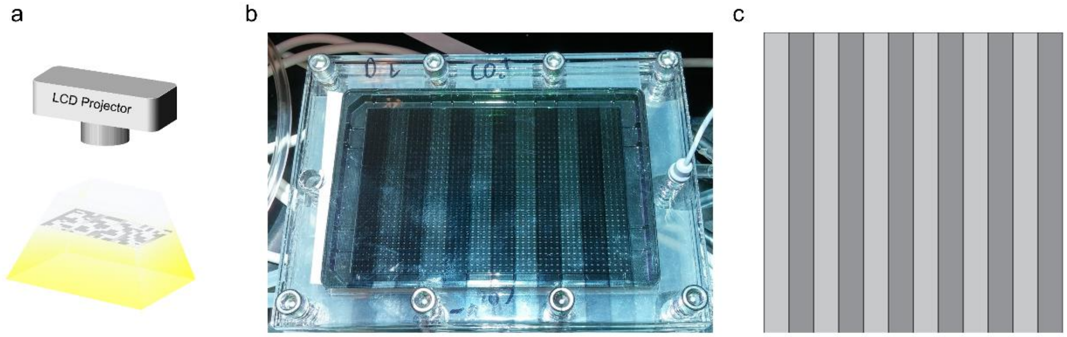

Figure S4: (a) Projection set up. The LCD projector is used to generate a light pattern over a well plate to control the light intensity in the well. Light intensity measurements (through the bottom of a black-walled well plate) were taken under uniform illumination to ensure that there was no spatial bias in light intensity. (b) Assembled gas gradient generator with 1536-well plate and experimental light pattern projected. The bright lanes correspond to an intensity of 60 μmol·m^{-2}s^{-1} while the dark lanes correspond

to an intensity of 30 µmol·m^{-2}s^{-1}. (c) Image used to project light pattern for 1536-well plate experiments. The bright lanes correspond to an intensity of 60 µmol·m^{-2}s^{-1} while the dark lanes correspond to an intensity of 30 µmol·m^{-2}s^{-1}.

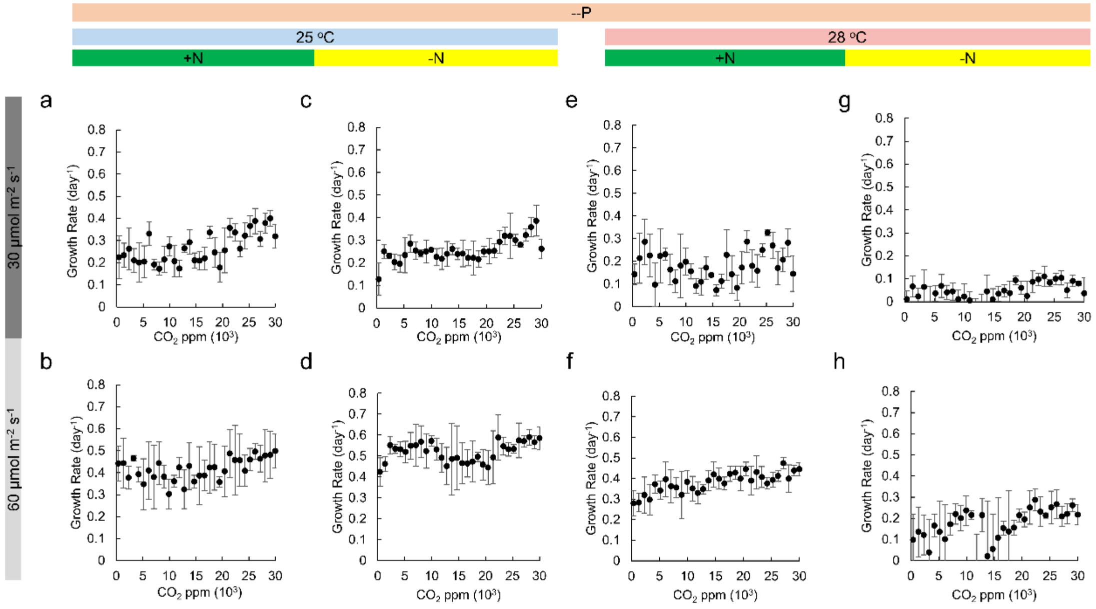

Figure S5: CO_2 response curves at 0.675 mM phosphorus showing the interactive effect of CO_2 (0-30 000ppm), Temperature (25 °C and 28 °C), Irradiance (30 µmol·m^{-2}s^{-1} and µmol·m^{-2}s^{-1}), and nitrogen (9.4 mM and 0.94 mM) In all cases n = 4 with error bars representing standard deviations.

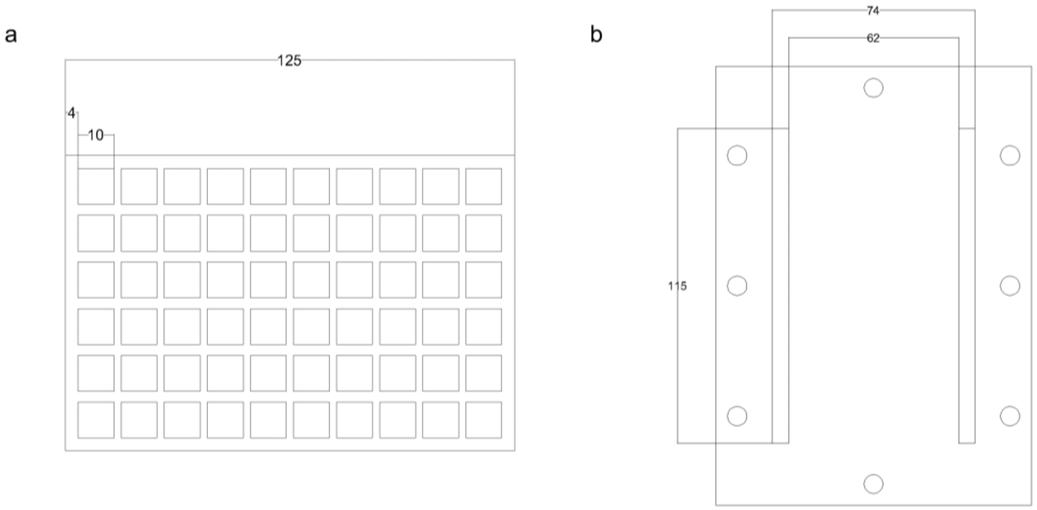

Figure S6: (a) Design of custom 60-well plate used for *Lemna gibba* experiments. All dimensions are in mm. The plate was fabricated from 4 layers of 3 mm thick PMMA. (b) Channel layout. All dimensions are in mm.

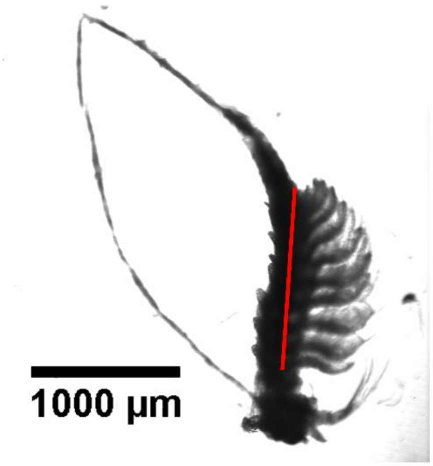

Figure S7: Measurement of brine shrimp abdominal length. Red line indicates length measurement.

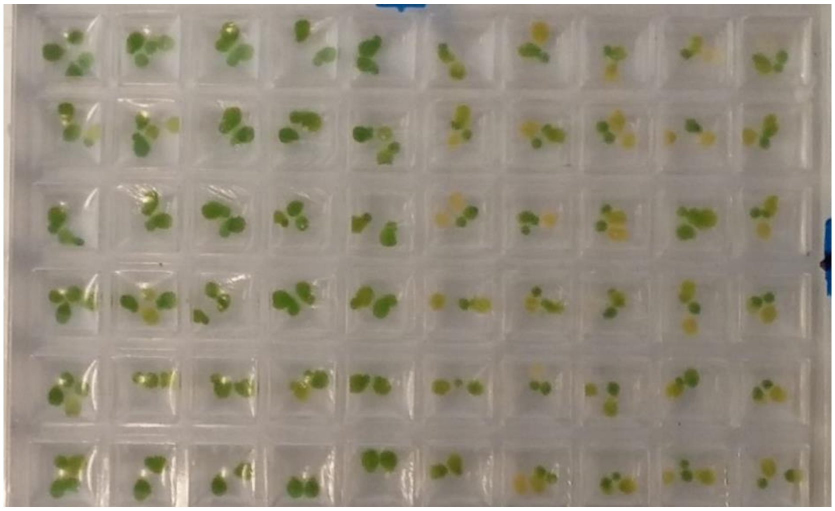

Figure S8: Photograph of an entire 60-well plate of *Lemna gibba* after 7 days of growth. The left half was subject to standard Hoagland's solution while the right half was subject to Hoagland's solution with LAS. A CO_2 gradient ranging from 400 ppm (bottom) to 4500 ppm (top) was applied.

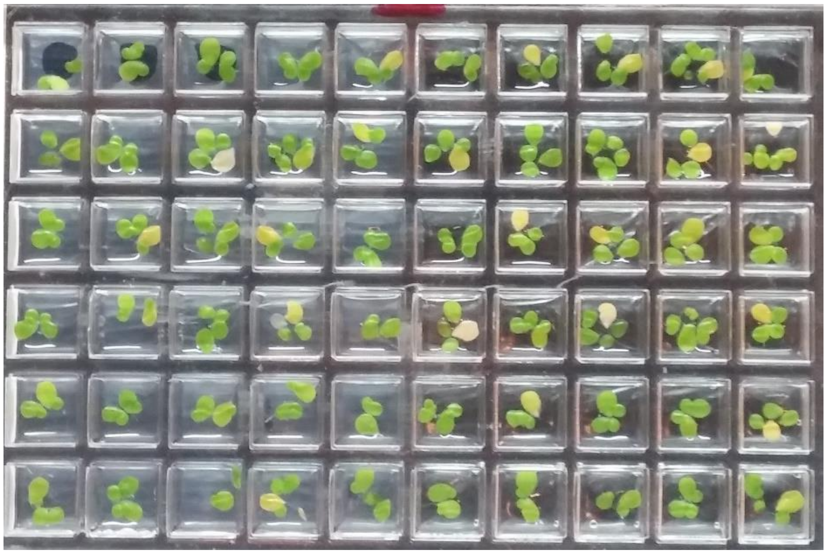

Figure S9: Photograph of an entire well plate of *Lemna Gibba* after 7 days of growth. The right half was subject to standard Hoagland's solution while the left half was subject to Hoagland's solution with titania nanoparticles. A CO_2 gradient ranging from 400 ppm (bottom) to 4500 ppm (top) was applied.

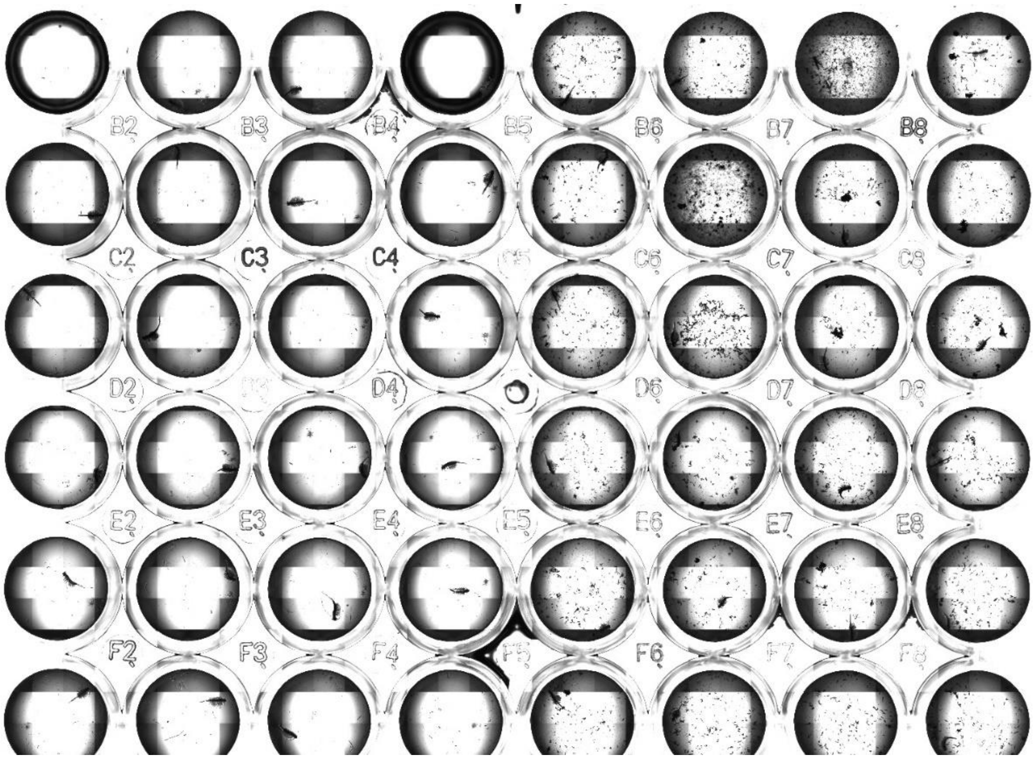

Figure S10: A full well plate of *Artemia Salina* after 7 days of growth from freshly hatched nauplii. The right half was subject to brine with titania nanoparticles while the left half was subject to regular brine. A CO_2 gradient ranging from 400 ppm (top) to 4500 ppm (bottom) was applied.

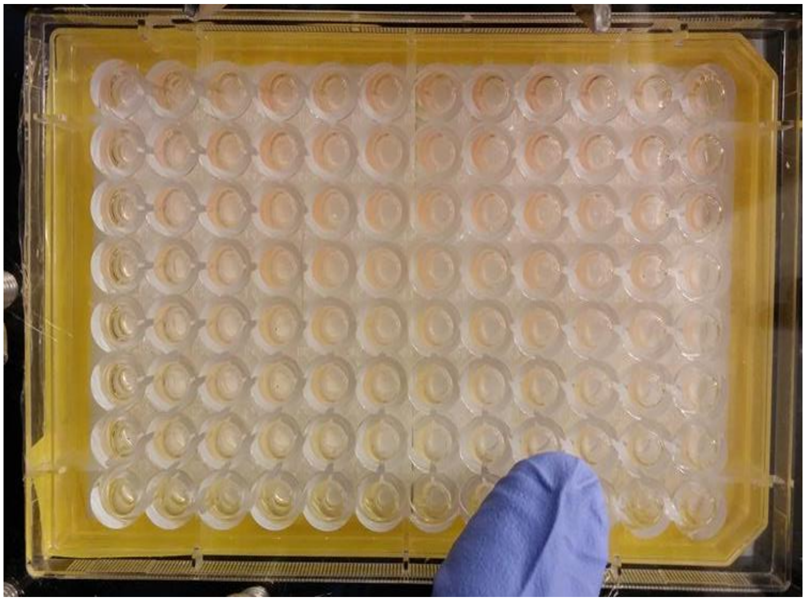

Figure S11: Methyl red indicator test. Methyl red changes from yellow to red in response to lower pH (higher CO_2).

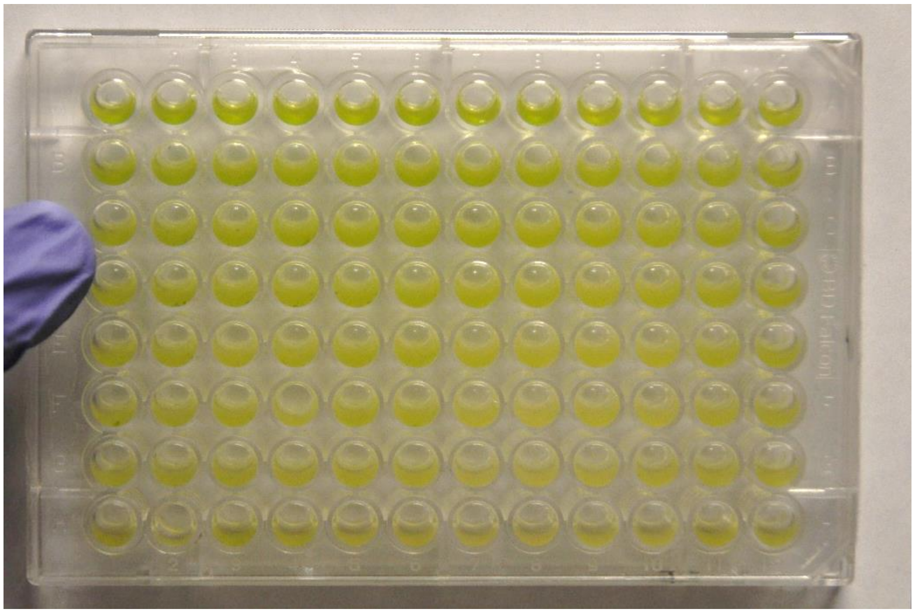

Figure S12: Illustration of crosstalk. This clear 96-well plate was subject to an image with 3 light levels (and 8 CO_2 levels) without changing other parameters. However, because of the clear walls cross talk between the wells created a more continuous light gradient and corresponding growth response.

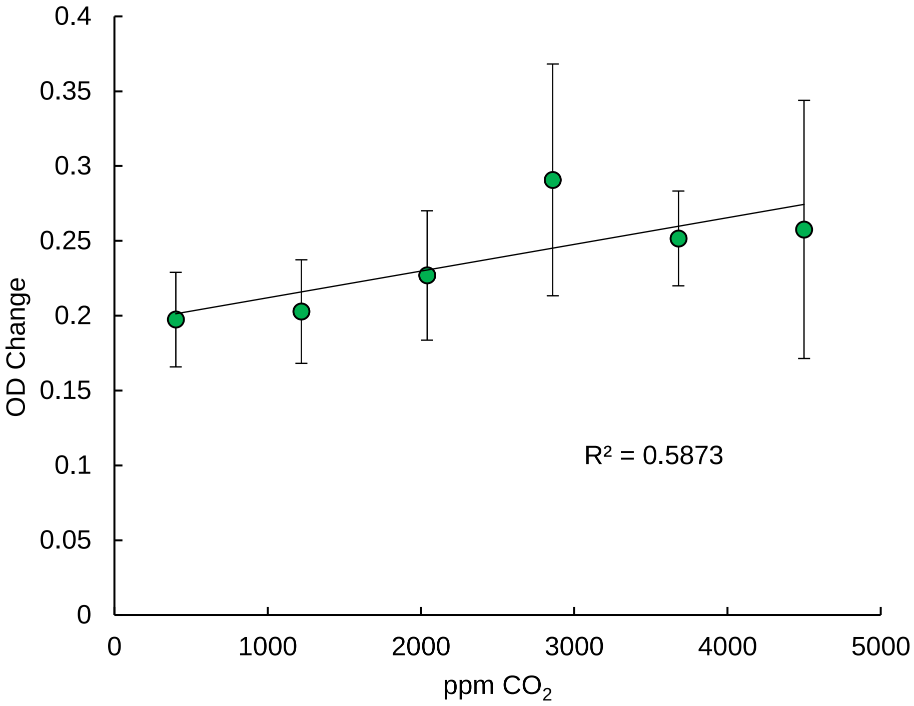

Figure S13: Growth of *Duniellia salina* under different concentrations of CO_2, line represents linear regression. Error bars are s.d. of n = 8 cultures.

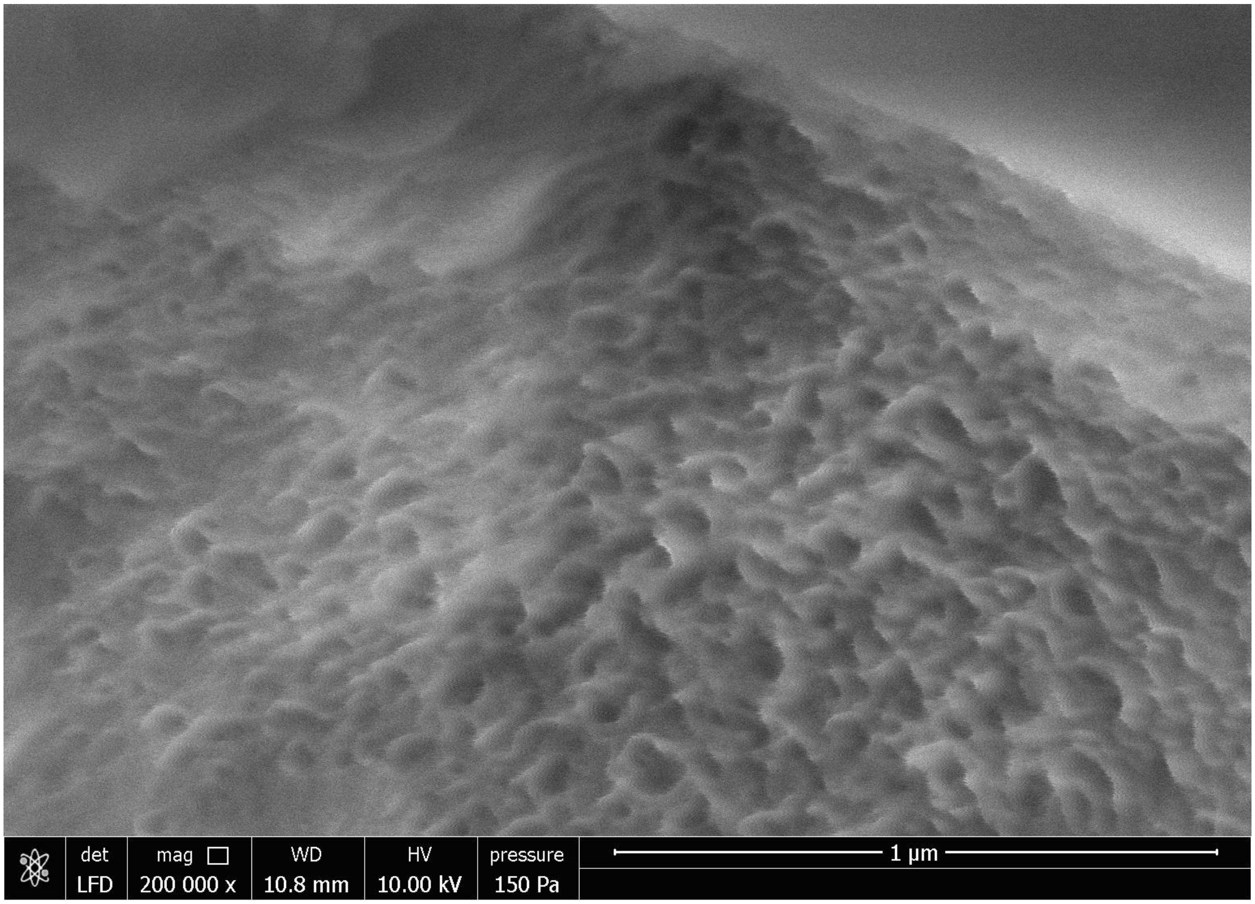

Figure S14: SEM image of the aerogel showing pore sizes of ~50nm.

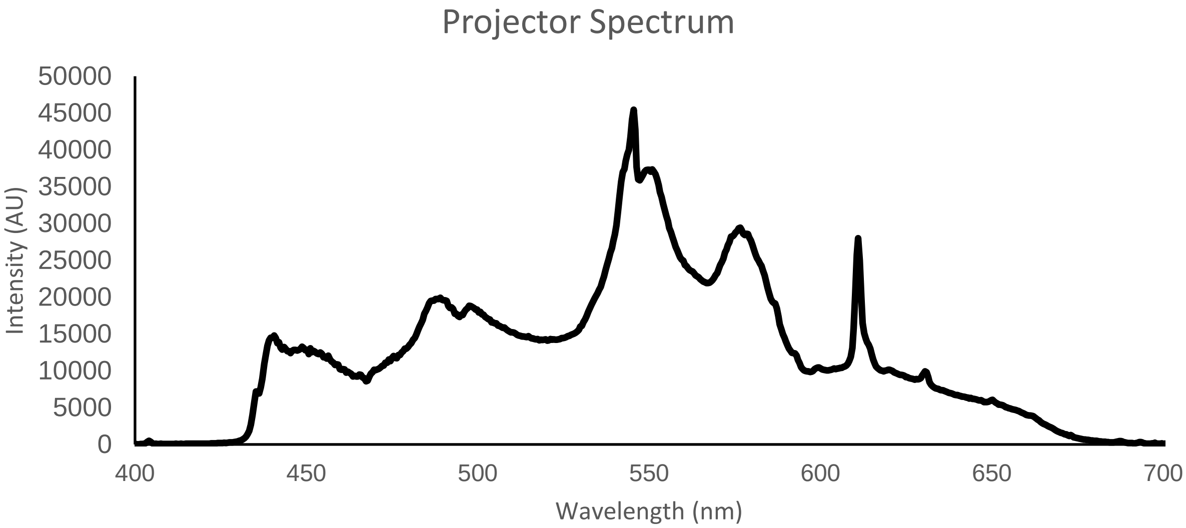

Figure S15: Color spectrum of projector output.

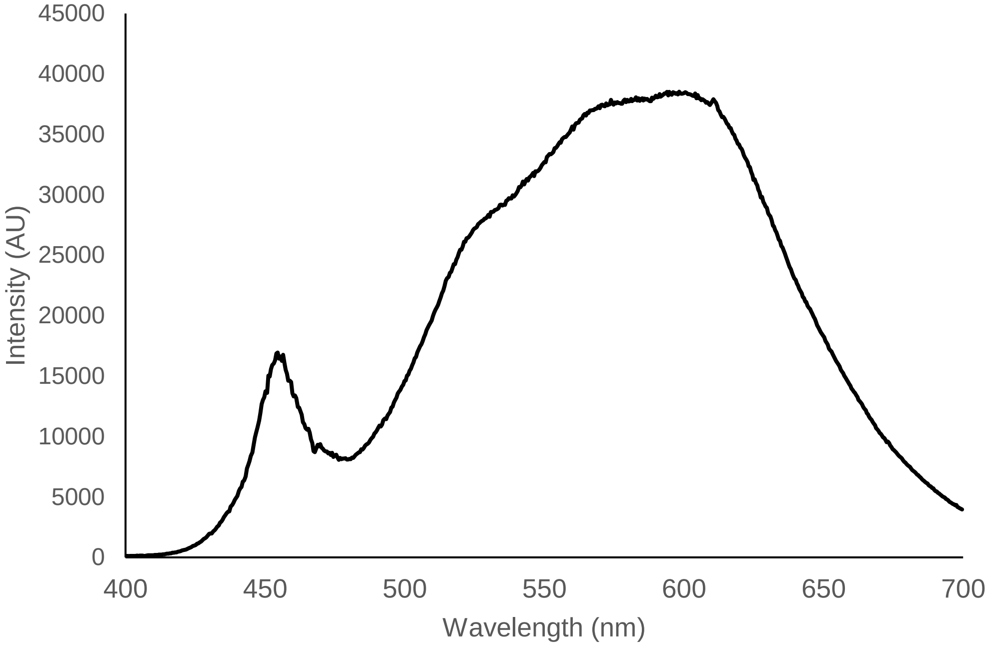

Figure S16: Color spectrum of LED output.

References

[1] M. P. Davey, I. Horst, G. H. Duong, E. V. Tomsett, A. C. P. Litvinenko, C. J. Howe, A. G. Smith, *Eukaryot. Cell* **2014**, *13*, 392.

[2] D. F. Sueltemeyer, K. Klug, H. P. Fock, *Plant Physiol.* **1986**, *81*, 372.

[3] G. Amoroso, D. Su, C. Thyssen, H. P. Fock, *Plant Physiol.* **1998**, 116, 193.
